# Comparison of the choice of animals for re-sequencing in two maternal pig lines

**DOI:** 10.1186/s12711-022-00706-w

**Published:** 2022-02-19

**Authors:** Christina M. Dauben, Christine Große-Brinkhaus, Esther M. Heuß, Hubert Henne, Ernst Tholen

**Affiliations:** 1 Institute of Animal Science, University of Bonn, Endenicher Allee 15, 53115 Bonn, Germany; 2 BHZP GmbH, An der Wassermühle 8, 21368 Dahlenburg-Ellringen, Germany

## Abstract

Next-generation sequencing is a promising approach for the detection of causal variants within previously identified quantitative trait loci. Because of the costs of re-sequencing experiments, this application is currently mainly restricted to subsets of animals from already genotyped populations. Imputation from a lower to a higher marker density could represent a useful complementary approach. An analysis of the literature shows that several strategies are available to select animals for re-sequencing. This study demonstrates an animal selection workflow under practical conditions. Our approach considers different data sources and limited resources such as budget and availability of sampling material. The workflow combines previously described approaches and makes use of genotype and pedigree information from a Landrace and Large White population. Genotypes were phased and haplotypes were accurately estimated with AlphaPhase. Then, AlphaSeqOpt was used to optimize selection of animals for re-sequencing, reflecting the existing diversity of haplotypes. AlphaSeqOpt and ENDOG were used to select individuals based on pedigree information and by taking into account key animals that represent the genetic diversity of the populations. After the best selection criteria were determined, a subset of 57 animals was selected for subsequent re-sequencing. In order to evaluate and assess the advantage of this procedure, imputation accuracy was assessed by setting a set of single nucleotide polymorphism (SNP) chip genotypes to missing. Accuracy values were compared to those of alternative selection scenarios and the results showed the clear benefits of a targeted selection within this practical-driven approach. Especially imputation of low-frequency markers benefits from the combined approach described here. Accuracy was increased by up to 12% compared to a randomized or exclusively haplotype-based selection of sequencing candidates.

## Background

Next-generation sequencing (NGS) experiments provide data that are of increasing importance in animal genetics with the potential to improve knowledge of the genetic background of complex traits with societal and economical interest (e.g., [1]). In addition, human genetics can benefit from insights into the porcine genome due to physiological similarities [2].

Until now, many of the genetic analyses in pigs are based on single nucleotide polymorphism (SNP) genotype data from chip arrays (e.g., [3, 4]). However, as costs for re-sequencing decrease [5], NGS becomes increasingly important [6]. The ratio between genetic gain and costs needs to be considered [7], as costs are a central issue in pig breeding [8].

Available NGS data are used to enhance information content of routinely phenotyped and genotyped animals, especially in terms of imputation from a lower to a higher marker density. An efficient approach is to select a highly informative and representative subset of animals for re-sequencing and to use it as reference panel for imputation of a population that is genotyped at a lower marker density (e.g., [9]). Several studies have described how the final selection criteria were determined for selecting the animals to be resequenced, e.g., how frequently they are used for artificial insemination and the availability of sampling material or special phenotypes (e.g., [10–12]). Within the 1000 Bull Genomes Project, selection based on pedigree-based algorithms has been described [13, 14].

In a simulated data set, five approaches to select candidates for re-sequencing were discussed by Druet et al. [13]. Results were evaluated by considering minor allele frequency (MAF) and effects on imputation accuracy and genomic prediction [13].

NGS data and imputed whole-genome sequence data are assumed to include causative variants and to enable their detection (e.g., [1, 14, 15]). In this context, the composition of the resequenced subset is of high importance [15]. Verification, validation and fine-mapping of quantitative trait loci (QTL) derived from genome-wide association studies can benefit from imputed sequence data [14], although the relatively high level of linkage disequilibrium in pig breeding populations makes detection of causative variants more difficult. Combining statistical, bioinformatic, and functional information, such as genetic associations, linkage disequilibrium, annotation, and functional genomic data, might optimize such detection (e.g., [16–18]) and, in addition, will increase the number of QTL and the proportion of variance explained by these QTL, compared to the use of a lower marker density [19].

The aim of this study was to evaluate alternative strategies for the choice of animals for re-sequencing in the maternal Landrace (LR) and Large White (LW) pig lines. Selection was conducted by applying several theoretical concepts and approaches under practical conditions. Selection steps were built on existing pedigree and real SNP genotype information within the pigFit project which focuses on piglet survival and immunocompetence [20, 21]. Performance of the different selection strategies was assessed based on the accuracy of imputation of masked genotypes from SNP chip data. The resulting NGS data are expected to enable a highly accurate imputation within the LR and LW population and to discover the causative genetic variants for previously detected QTL regions.

## Methods

### Animals and data sets

The aim of the project ’pigFit-Molecular genetic and immunological analysis of survival and postnatal growth of piglets’ is to identify biological relevant regions associated with survivability, health and immune traits in piglets and growing pigs. Within this project, phenotyping and genotyping were performed in the two maternal pig lines LR and LW. Blood and tissue samples were collected from piglets and their biological dams. SNP genotype data, based on the PorcineSNP60v2 BeadChip (Illumina Inc., San Diego, CA, USA), were used to conduct the animal selection process. In total, SNP genotype information for 944 LR and 800 LW animals that were genetically linked to the pigFit data set was available. Before starting the analysis, breed-specific quality control was performed for SNPs on autosomes. Markers showing a high linkage disequilibrium ($r^2>$0.8) within a region of 3 kb and markers with a low MAF (<1%) or a low call rate (<95%) were excluded. In addition, breed-specific pedigree information for 2871 LR animals and 1965 LW animals was available.

### Theoretical principles for selection of animals and implementation

Druet et al. [13] have explained the impact of different strategies to choose individuals for sequencing on imputation accuracy and genomic prediction based on simulated sequence data. These approaches can be summarized as pedigree-based, haplotype-based, and randomized selection of individuals.

Based on the principles and results presented by Druet et al. [13], the current study applied a combined, heuristic approach (C) to select animals for re-sequencing. This approach combined haplotype- and pedigree-based methods according to their theoretical benefits, which are described below.

#### Random selection

Compared to targeted selection, random selection has been demonstrated to be a non-competitive approach [13]. In the current study, random selection of animals for sequencing was used for comparative purposes in validation steps.

#### Haplotype-based (H) approach

Haplotype-based selection aims at maximizing the diversity of haplotypes with or without weighting the haplotype frequency [13]. Our haplotype-based selection strategy made use of 60k SNP genotype information. SNP genotypes were processed using the software AlphaPhase [22] to construct haplotypes by phasing. Phasing was performed by breed and chromosome. Chromosomes were divided into fragments of 100 consecutive SNPs (*GeneralCoreLength*), as recommended for a 60k SNP density [22]. Hickey et al. [22] demonstrated that the highest percentage of alleles correctly phased were obtained for this setting. The average percentage of genotypes phased was >99%, indicating that phased genotypes can be used in subsequent analyses.

In general, the aim of the H approach is to cover the maximum proportion of haplotype diversity within the population. The results obtained with simulated data led to the assumption that these animals will enable accurate imputation from chip genotype data to sequence data [13].

To carry out the selection step, we used the AlphaSeqOpt-Method 1 [23]. In a nutshell, haplotype libraries were constructed and animals with the most high-frequency haplotypes in the investigated population were identified as candidates for re-sequencing to enable exact and complete imputation [23]. Appropriate haplotypes were masked and 100-fold repetition was performed.

The settings for the AlphaSeqOpt software were modified according to the porcine genome structure with 18 autosomes and to the chip structure with a varying number of SNPs per chromosome. The call rate of SNPs and individuals had to exceed 90% to be included in the construction of the haplotype library. Information on the number of cores and the core length was adapted to the *GeneralCoreLength* used in the AlphaPhase software. Since the H approach aimed at covering a large amount of haplotypes, the threshold for haplotype frequency in the population was set to 0 to take all the haplotypes into account, regardless of their frequency. In both lines, the availability of sample material was a key limiting factor, particularly in previous generations. Thus, the target number of animals to select for re-sequencing was set to 100. Animals were added to the candidate set in an iterative manner. Although the AlphaSeqOpt software includes an additional approach, Method 2 [24], this method was not implemented because of uncertainties in defining the cost parameters due to technical innovations and because of known difficulties in the analysis of specific regions in the porcine genome.

#### Pedigree-based (P) approaches

One of the strategies discussed by Druet et al. [13] maximizes the expected genetic relationship between sequenced animals and the population based on pedigree data. For implementing the P approach in our study, the AlphaSeqOpt software (P1) [23] was used and the algorithm was as described by Goddard et al. [25]. Basic settings were comparable with those in the H approach but the parameter *OptimisationMethod* was set to *Pedigree*. A subset of 100 candidates from the genotyped animals was selected based on pedigree relationships [23, 25].

The ENDOG v.4.8 software [26] was used to determine the underlying population structure and to characterize each population. Unlike all other approaches, this software makes use of the entire pedigree of the pigFit population and available relatives, covering 17 generations. *Ancestors* (P2) were identified as the animals that best explained the genetic variability of both populations considering their unbalanced use in breeding [26, 27].

#### Selection of animals for re-sequencing by the combined (C) approach

As the starting point for the C approach, all animals from the H, P1, and P2 approaches were taken into account to select animals for re-sequencing. According to the available budget, the target number of animals was set to 57, divided into 28 LR and 29 LW animals. The C approach is practical-driven and aims at optimizing imputation accuracy of common and low-frequency variants by combining haplotype- and pedigree-based criteria. The findings shown by Druet et al. [13] provided an indication for the prioritization between the three approaches. As a consequence, we defined steps 1 to 4 for the final selection of candidates for re-sequencing. These steps were conducted successively and included the availability of sample material as a requirement.

- Step 1: Animals that were selected by the H approach *and* at least by one of the P approaches (P1, P2)
- Step 2: Animals that were selected in both P approaches (P1, P2)
- Step 3: Animals that were selected by the H approach *and* both parents, sire and dam, were identified as important ancestors (P2)
- Step 4: Animals that were selected by the H approach *and* one parent was identified as an important ancestor (P2)

#### Validation

**Fig. 1:**
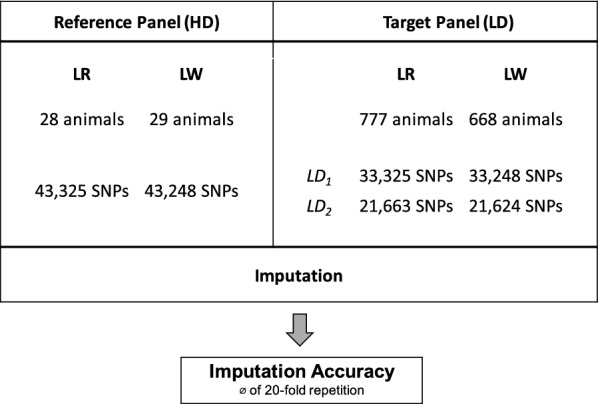
Workflow for the evaluation of imputation accuracies. Number of animals and SNP genotypes after quality control included in imputation steps; HD: high marker density, LD: low marker density, LR: Landrace, LW: Large White, LD_1_: Imputation scenario 1 (Imputation of 10,000 SNPs), LD_2_: Imputation scenario 2 (Imputation of 50% of the SNP set)

Evaluation of alternative selection strategies was performed based on the 60k SNP genotype data in a 0, 1, 2 coding as obtained from the PorcineSNP60v2 BeadChip (Illumina Inc., San Diego, CA, USA). The workflow for the validation is shown in Fig. 1. Breed-specific data sets were split into a reference panel with high marker density (HD) and a target panel with low marker density (LD). The reference panel with high marker density included 28 LR animals and 29 LW animals. Three sets of candidates for re-sequencing were analyzed. The set comprising candidates selected by the C approach (*combined sample*) was compared to the set of the top candidates from the H approach (*haplotype sample*) and a set of randomly selected animals in a 30-fold repetition (*random sample*). Genotype data in the reference panel contained information on all markers that passed quality control (LR n_SNPs_=43,325, LW n_SNPs_=43,248).

The remaining animals (LR n=777, LW n=668) were allocated to the target panel. The assessed imputation scenarios are shown in Fig. 1. LD genotype data was simulated by setting 10,000 SNPs/ 23.1% (LD_1_) or half of the number of SNPs (LD_2_) to missing. Imputation was performed from LD to HD for each of the reference panels *combined sample*, *haplotype sample*, and *random sample*, using FImpute v3 [28]. Imputation accuracy was calculated as the correlation (r) between true and imputed genotypes. Mean accuracies were averaged over a 20-fold repetition of imputation.

## Results and discussion

### Haplotype-based (H) approach

**Fig. 2:**
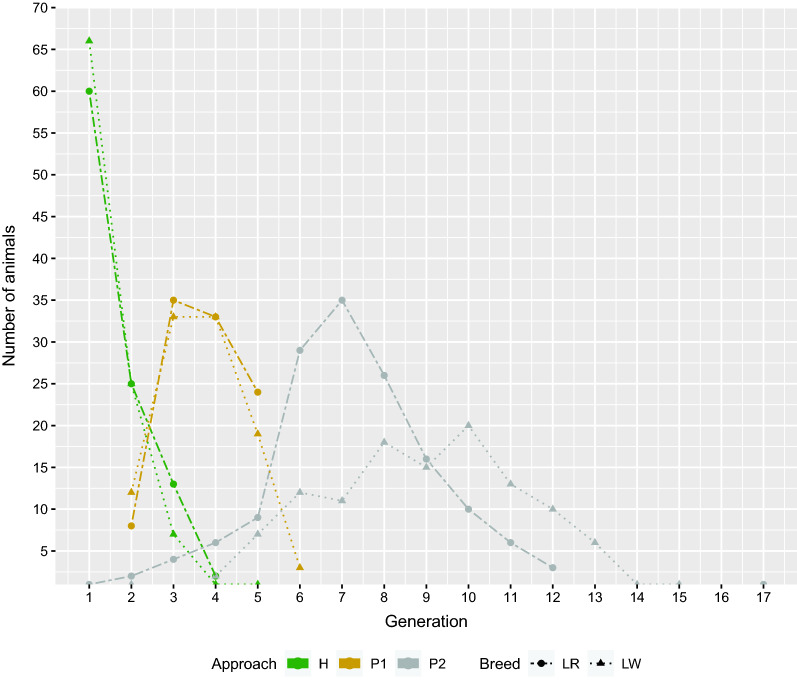
Distribution of candidates among generations and breeds. Results from the H approach n = 100, P1 approach n = 100 and P2 approach n = 148(LR)/117(LW); Generation 1: most recent generation

The H approach resulted in 100 candidates per breed that maximized the diversity of haplotypes across the population. As shown in Fig. 2, in LR, 60% of the candidates belonged to the most recent generation. The remaining 40% were distributed among the three previous generations, with decreasing proportions. In LW, the proportion of animals in the most recent generation was slightly higher (66%). The remaining candidates had a distribution similar to that for LR.

### Pedigree-based (P) approaches

The P1 and H approaches are based on the same animals originating from the most recent generations. Within the P1 approach, generations 3 and 4 (relative to the most recent generation being generation 1) comprised about two thirds of the 100 candidates in each breed (see Fig. 2).

The P2 approach included 17 generations of pedigree for both populations. In total, 148 LR and 117 LW animals were selected based on pedigree relationships. Selected LR animals were from 13 of the 17 generations, with the largest number from generations 6 to 8 (see Fig. 2), which were not selected by the H and P1 approaches. LW candidates were more equally distributed in generations 6 and older.

**Fig. 3:**
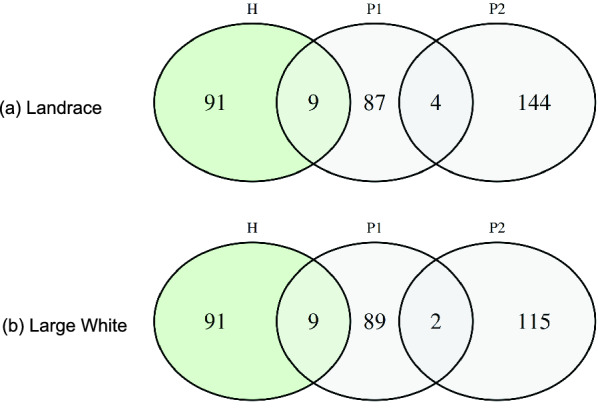
Venn diagram of animals selected by the haplotype-based (H) and pedigree-based (P) approaches. Total number of animals per method: H approach n = 100, P1 approach (AlphaSeqOpt [25]) n = 100, P2 approach (ENDOG v.4.8 [26]) n = 148(LR)/117(LW)

Concordance between the two P approaches was observed for four LR animals and two LW animals (see Fig. 3). Strongly diverging results between the P1 and P2 approaches were expected due to the differences in databases and purposes of the methods. The P1 approach selected animals by focusing on the pedigree relationships in genotyped animals of recent generations, while the P2 approach identified animals that explained the genetic variability of both populations, based on pedigree information going back 17 generations.

### Subset of animals for re-sequencing selected by the combined (C) approach

Figure 3 provides the number of selected animals from the H, P1, and P2 approaches. These results were condensed to the target number of 57 animals within our C approach with respect to optimizing imputation.

Selection steps 1 and 2 (see “Methods” section) are the overlaps of two approaches. Step 1 (H $\cap$ P1 or H $\cap$ P2) is based on both, haplotype- and pedigree-based approaches, whereas step 2 (P1 $\cap$ P2) is solely based on pedigree-based criteria. In total, steps 1 and 2 identified nine and four LR animals as well as nine and two LW animals for re-sequencing, respectively. In order to complete the target number, steps 3 and 4 were implemented. The resulting animals fulfilled the haplotype-based criteria and were progeny of animals which were selected by one of the pedigree-based approaches. In comparison to steps 1 and 2, the importance of the pedigree-based criteria is downgraded in steps 3 and 4, but still effective in the representation of the diversity within the LR and LW populations. The resulting subset of animals is hereafter denoted as *combined sample*.

The *combined sample* included 28 LR and 29 LW animals, divided into 30 males and 27 females. These animals were born between 2012 and 2016 and can be assigned to six generations (see Table 1).

**Table 1:** Distribution among breeds and generations of animals selected for re-sequencing using the combined (C) approach

Generation	1	2	3	4	5	6
Landrace	8	8	7	4	1	0
Large White	8	12	5	2	1	1

Generation 1: most recent generation of animals

### Validation

Imputation accuracy was calculated to evaluate and quantify the performance of the selected animals for re-sequencing in a practical data set based on 60k SNP genotypes. The detailed results are in Table 2 with MAF grouped in the classes 1 to 3%, 3 to 5% and >5%. In summary, imputation accuracy was high using the *combined sample* as the reference panel. The mean accuracy ranged from 83.6 to 93.1% in LR and from 86.2 to 93.9% in LW across both imputation scenarios (LD_1_, LD_2_) and all MAF classes (1–3%, 3–5%, >5%). Imputation using the *random sample* (LR 74.1–87.8%, LW 78.7–89.9%) and *haplotype sample* (LR 81.2–94.1%, LW 83.4–94.0%) as reference panel had a lower mean accuracy.

**Table 2:** Imputation accuracy (r) of masked chip genotype data from lower (LD_1_, LD_2_) to higher marker density in Landrace (LR) and Large White (LW) using different reference panels

Breed	Reference panel	Imputation scenario	MAF 1–3%			MAF 3–5%			MAF >5%		
			$\varnothing$	Min.	Max.	$\varnothing$	Min.	Max.	$\varnothing$	Min.	Max.
LR	com	LD_1_	86.53%	83.97%	88.95%	86.30%	84.51%	87.73%	86.30%	84.51%	87.73%
		LD_2_	83.63%	82.26%	85.23%	83.60%	82.00%	85.77%	93.13%	92.88%	93.29%
	ran	LD_1_	74.13%	63.52%	84.26%	75.86%	66.82%	81.95%	86.81%	71.26%	88.66%
		LD_2_	75.35%	70.80%	80.38%	77.65%	73.68%	81.18%	87.83%	87.23%	88.28%
	hap	LD_1_	82.81%	80.38%	85.43%	84.64%	81.24%	86.83%	94.10%	93.88%	94.52%
		LD_2_	81.21%	78.81%	83.31%	82.14%	80.74%	84.30%	92.98%	92.79%	93.20%
LW	com	LD_1_	92.68%	92.47%	92.85%	88.68%	86.37%	89.97%	93.94%	93.70%	94.16%
		LD_2_	86.52%	85.22%	88.23%	86.20%	84.93%	87.47%	92.71%	92.59%	92.90%
	ran	LD_1_	83.30%	76.37%	88.20%	82.24%	76.15%	86.73%	89.90%	77.65%	90.86%
		LD_2_	79.22%	73.35%	83.88%	78.68%	74.33%	83.88%	88.35%	87.41%	88.94%
	hap	LD_1_	84.59%	81.45%	87.05%	87.36%	85.79%	89.46%	93.97%	93.77%	94.17%
		LD_2_	83.37%	81.34%	85.35%	85.46%	84.05%	86.54%	92.86%	92.66%	93.03%

Imputation accuracy (r): correlation between true and imputed genotypes, com: *combined sample*﻿, ran: *random sample*, hap: *haplotype sample*, LD_1_: 10,000 SNPs (23.1%) were set to missing, LD_2_: 50% of the SNPs were set to missing, MAF: Minor allele frequency

Assessment of the imputation accuracy was performed by imputing masked genotypes from the PorcineSNP60v2 BeadChip (Illumina Inc., San Diego, CA, USA) in a 20-fold repetition and reflect the accuracy of imputation to a HD density in these populations. However, this study cannot make a conclusive statement regarding the accuracy for imputation to sequence data.

Overall, imputation of 10,000 masked SNPs (LD_1_) led to higher accuracies compared to imputation of 50% of the SNP set (LD_2_). Exceptions were found for the *random sample* in LR and the MAF class >5% in the *combined sample* in LR.

The strategy for selecting animals is very important, especially to enable an accurate imputation of low-frequency variants [13]. As expected from previous studies (e.g., [13, 29]), imputation tended to be more accurate as the MAF of markers increased. Although imputation of low-frequency markers is less accurate (e.g., [13, 29]), imputation using the *combined sample* as the reference panel reached the highest increase in accuracy for low-frequency markers, compared to the alternative reference panels (*haplotype sample, random sample*). This may be due to the prioritization of haplotype diversity in the selection process for the *combined sample* and to the imputation to 60k level. However, in terms of low-frequency variants, imputation to the sequence level remains a major challenge, especially with a small number of sequenced animals.

In addition, our study observed differences in imputation accuracy between LR and LW. For low-frequency (1–3%) and higher-frequency markers (>5%), imputation performance was better in LW. In addition to breed-specific genetic aspects, one possible explanation is the larger number of animals within the reference panel for LW. It is important to underline that both nucleus populations are genetically distinct and differ in their breeding objectives to a considerable extent. Moreover, genotyped animals were assumed to be representative samples of both nucleus populations.

**Fig. 4:**
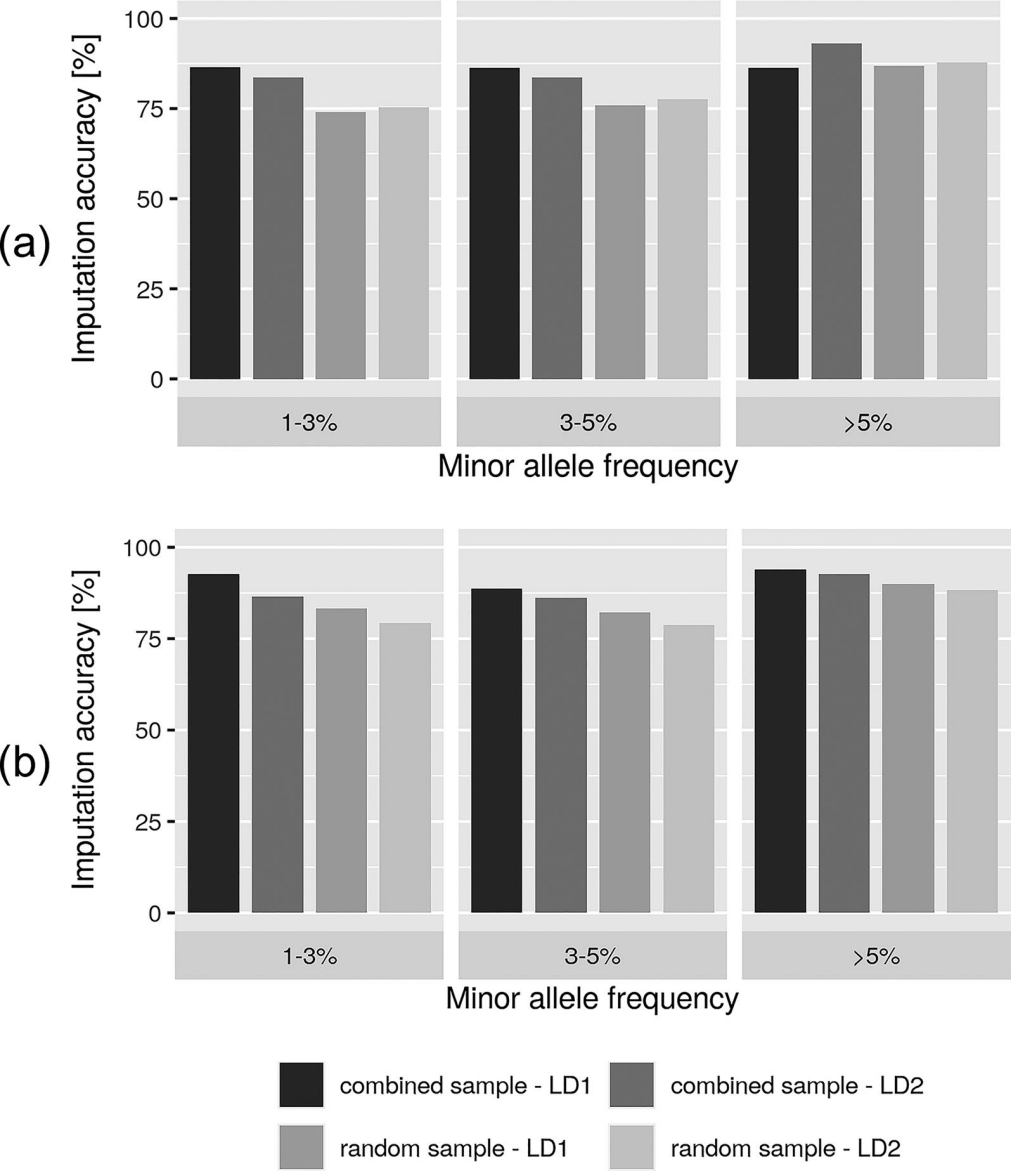
Imputation accuracy using the combined sample and the random sample as reference panels in Landrace (**a**) and Large White (**b**)

Compared to random selection, targeted selection approaches achieved up to 12.4% higher imputation accuracy using the *combined sample* as reference panel for LR (see Fig. 4). The highest increase in imputation accuracy compared to random selection was reached with markers that had a MAF of 1 to 3%. The advantage of the *combined sample* compared to the *random sample* was more evident for LR than for LW except for markers with a MAF >5% in LD_1_ and a MAF of 3 to 5% in LD_2_. The gain in accuracy through targeted selection was mainly achieved when imputing a larger number of markers (LD_2_).

In general, imputation accuracy varied a lot between replicates of randomly selected reference panels. This emphasizes the importance of the structure of reference panels for re-sequencing experiments, especially when resources are limited [15]. Our results are consistent with those of Druet et al. [13], who showed the advantages of haplotype-based selection over randomized selection in a simulated data set.

Assuming the availability of sample material from all prioritized candidates, the H approach was tested as an alternative approach, by considering haplotype-based criteria, only. It should be noted that the C approach is a heuristic, practical-driven approach and its results differ slightly from those using exclusively the H approach. For markers with a higher MAF (>5%), we observed no improvement in imputation accuracy of the C approach compared to the H approach. The latter achieved an even slightly higher accuracy for LD_1_ in LR. Nevertheless, an increase of about 2.4 to 8.1% was found with low-frequency markers of the C over the H approach.

Targeted selection using the C approach with haplotype- and pedigree-based selection criteria, as described in this paper, shows that it is superior compared to alternative approaches to select animals for a re-sequencing experiment using data from the LR and LW populations investigated here.

The respective characteristics of the LR and LW populations are common for commercial maternal nucleus breeding populations. Moreover, we had to consider the practical constraints (data and sample availability, and budget, which lead to a trade-off between the number of animals and coverage) within our C approach. Against this background, our practical-driven approach and its results could be considered for similar situations in pig breeding using the data of nucleus herds.

Data from re-sequencing the *combined sample* will be used to supplement the pigFit project in future investigations. Making use of highly informative genotype data, genome-wide association studies (GWAS) that use highly informative genotypes are a highly efficient approach to map loci and to detect QTL and candidate genes for complex traits (e.g., [1]). This approach enables fine mapping [19] and validation of previously identified regions in a GWAS for important traits (e.g., [21]).

## Conclusions

Targeted animal selection for re-sequencing is recommended. Taking into account all available information on population structure, when selecting animals for re-sequencing, including pedigree information and additional SNP genotype information, increased the imputation accuracy of masked SNP chip genotypes, measured as the correlation between true and imputed genotypes, by an average of around 7.3 to 12.4% for low-frequency markers and up to 5.3% for markers with a MAF >5%. The combined use of pedigree- and genotype-based approaches in practical data sets is particularly suitable for improving imputation accuracies for low-frequency markers.

## Data Availability

Data cannot be made publicly available, as they are owned by a third party, the BHZP GmbH. The data sets used and analysed during the current study are available from the corresponding author on reasonable request and with permission of the BHZP GmbH pig breeding company (henne@bhzp.de).
